# Overexpression of a microRNA-targeted NAC transcription factor improves drought and salt tolerance in Rice via ABA-mediated pathways

**DOI:** 10.1186/s12284-019-0334-6

**Published:** 2019-10-21

**Authors:** Dagang Jiang, Lingyan Zhou, Weiting Chen, Nenghui Ye, Jixing Xia, Chuxiong Zhuang

**Affiliations:** 1 State Key Laboratory for Conservation and Utilization of Subtropical Agro-bioresources, College of Life Sciences, South China Agricultural University, Guangzhou, 510642 China; 2 Laboratory Center of Basic Biology and Biotechnology, Education Department of Guangdong Province, Zhongkai University of Agriculture and Engineering, Guangzhou, 510225 China; 3 Southern Regional Collaborative Innovation Center for Grain and Oil Crops in China, College of Agriculture, Hunan Agricultural University, Changsha, 410128 China; 4 State Key Laboratory for Conservation and Utilization of Subtropical Agro-bioresources, College of Life Science and Technology, Guangxi University, Nanning, 530004 China

**Keywords:** Rice (*Oryza sativa*), *OsNAC2*, ABA, Drought tolerance, Salt tolerance, microRNA

## Abstract

**Background:**

The NAC (NAM, AFAT, and CUC) transcription factors play critical roles in rice (*Oryza sativa*) development and stress regulation. Overexpressing a microRNA (miR164b)-resistant *OsNAC2* mutant gene, which generates transcripts that cannot be targeted by miR164b, improves rice plant architecture and yield; however, the performance of these *mOsNAC2*-overexpressing lines, named ZUOErN3 and ZUOErN4, under abiotic stress conditions such as drought have not yet been fully characterized.

**Results:**

In this study, we showed that the germination of ZUOErN3 and ZUOErN4 seeds was delayed in comparison with the wild-type (WT) seeds, although the final germination rates of all lines were over 95%. The quantification of the endogenous ABA levels revealed that the germinating *mOsNAC2*-overexpressing seeds had elevated ABA levels, which resulted in their slower germination. The *mOsNAC2*-overexpressing plants were significantly more drought tolerance than the WT plants, with the survival rate increasing from 11.2% in the WT to nearly 70% in ZUOErN3 and ZUOErN4 plants after a drought treatment. Salt (NaCl) tolerance was also increased in the ZUOErN3 and ZUOErN4 plants due to significantly increased ABA levels. A reverse transcription quantitative PCR (RT-qPCR) analysis showed a significant increase in the expression of the ABA biosynthesis genes *OsNCED1* and *OsNCED3* in the *mOsNAC2*-overexpressing lines, and the expression levels of the stress-responsive genes *OsP5CS1*, *OsLEA3*, and *OsRab16* were significantly increased in these plants. Moreover, OsNAC2 directly interacted with the promoters of *OsLEA3* and *OsNCED3* in yeast one-hybrid assays.

**Conclusions:**

Taken together, our results show that *OsNAC2* plays a positive regulatory role in drought and salt tolerance in rice through ABA-mediated pathways.

## Background

Rice (*Oryza sativa* L.) is a major food crop worldwide, and as the population increases, so does the demand for this crop; however, rice is often exposed to drought, salt, and other stresses, which may severely affect its yield (Valliyodan and Nguyen, 2006). The cultivation of abiotic stress-resistant rice is therefore important for generating sufficient rice yields and ensuring food security.

To maintain growth under environmental stresses, plants often depend on hormones such as abscisic acid (ABA), which plays a critical role in integrating a wide range of stress signals and controlling the downstream stress responses (Zhu, 2016). ABA biosynthesis and the resulting endogenous ABA levels are increased under abiotic stresses, with drought and salt stresses representing the most important environmental signals upregulating the transcription of the ABA biosynthesis genes (Xiong et al., 2003).

The NAC (NAM, AFAT, and CUC) family of transcription factors (TFs) are plant-specific, and 151 of them have been detected in rice (Nuruzzaman et al., 2010). The rice NAC TFs play vital roles in diverse aspects of plant growth, development, and stress responses (Puranik et al., 2012). The involvement of the NAC TFs in rice abiotic stress responses has been extensively explored, and some rice *NAC* genes have been reported to function in abiotic stress tolerance; for example, the overexpression of *OsNAC5* (Takasaki et al., 2010; Song et al., 2011; Jeong et al., 2013), *OsNAC6* (Hu et al., 2008), *OsNAC9* (Redillas et al., 2012), *OsNAC10* (Jeong et al., 2010), or *OsNAP* (Chen et al., 2014) improves drought and salinity tolerance in transgenic rice.

Several studies have reported that *OsNAC2* has important functions in rice (Mao et al., 2007; Fang et al., 2014; Chen et al., 2015; Mao et al., 2017; Jiang et al., 2018). In a promoter activation-tagging mutant, an increase in *OsNAC2* expression results in an increased tiller angle in rice (Mao et al., 2007). In addition, *OsNAC2* is associated with plant height (Chen et al., 2015), the promotion of leaf senescence (Mao et al., 2017), and increased yields (Jiang et al., 2018), highlighting the important roles this gene plays in rice growth and development. Several NAC transcripts are cleaved by miR164 to modulate developmental processes (Mallory et al., 2004; Guo et al., 2005; Kim et al., 2009); for example, post-transcriptional modifications of the NAC domain TFs by miR164 function in the postembryonic regulation of the shoot meristem (Li and Zhang, 2016).

Some NAC TFs can improve abiotic-stress tolerance (Hu et al., 2006; Saada et al., 2013; Chen et al., 2014; Hong et al., 2016; Huang et al., 2016), with some even increasing yields under abiotic-stress conditions (Jeong et al., 2010; Redillas et al., 2012); however, there have been no reports of a single *NAC* gene regulated by a microRNA positively promoting plant architecture, yield, and abiotic-stress tolerance. Previously, we found that *OsNAC2* is a target of the microRNA miR164b (Jiang et al., 2018). When miR164b-resistant *OsNAC2* was overexpressed in rice, the transgenic plants exhibited ideotype plant architecture traits and had dramatically increased yields (Jiang et al., 2018); however, the performance of these transgenic plants in response to stress conditions has not yet been elucidated.

Here, we found that the plants overexpressing the miR164b-resistant form of *OsNAC2* had higher levels of drought and salt tolerance than the wild type (WT), and that the ABA content was increased in the transgenic plants. We also found that the expression levels of the ABA biosynthesis genes and the stress-responsive genes were increased in these lines relative to the WT. Our study provides new insights that may enable the development of high-yielding rice varieties with increased drought tolerance using genetic engineering.

## Results

### The overexpression of *OsNAC2* slows down germination

**Fig. 1:**
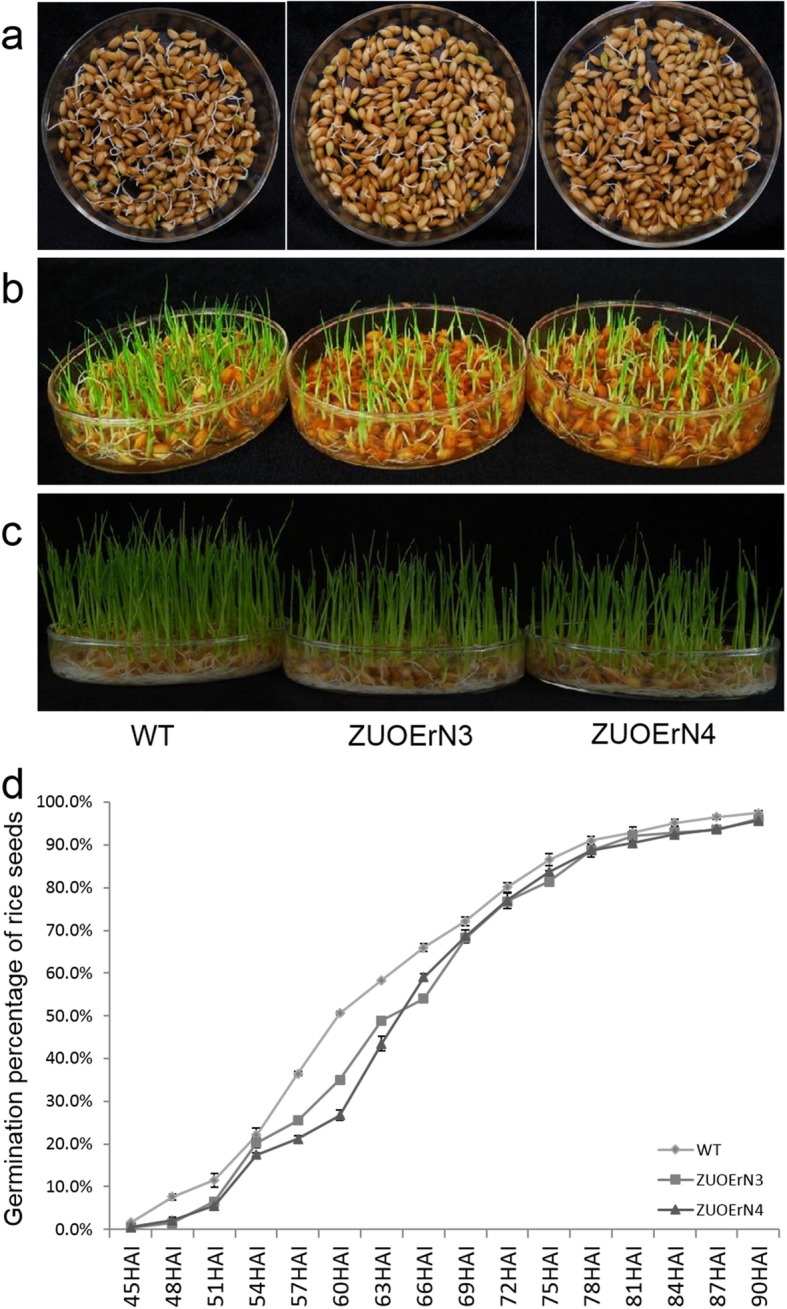
*mOsNAC2*-overexpressing seeds germinate more slowly than those of WT. The seeds were presoaked with distilled water for 48 h and then germinated at 25/23 °C (day/night) in petri dishes. The germination percentage was calculated at different times after imbibition. The images were taken at 3 d (**a**), 7 d (**b**), and 9 d (**c**) after imbibition in water. (**d**) Germination percentage after imbibition in water. WT: wild-type seeds; ZUOErN3 and ZUOErN4: *OsNAC2* overexpression lines; HAI: hours after imbibition. Error bars represent SD of three biological replicates (*n* > 200). ** *P* < 0.01 (*t*-test)

In our previous study, we showed the miR164b-resistant *mOsNAC2*-overexpressing lines, ZUOErN3 and ZUOErN4, had a significant high-yield potential (Jiang et al., 2018). In this study, we found that the germination of ZUOErN3 and ZUOErN4 seeds was substantially slower than that of the WT seeds (Fig. 1a–c). During the imbibition for three days, we observed that more seeds germinated from WT (80.1 ± 1.1%) than from the overexpressing plants (76.8 ± 0.3% and 76.9 ± 1.7% in ZUOErN3 and ZUOErN4, respectively; Fig. 1a, d). Five (Fig. 1b) and seven (Fig. 1c) days after germination, obvious differences were observed between the seedlings; for example, the WT seedlings were taller than the overexpression plants. Further observation after 48 h of imbibition showed that the germination percentage of the WT seeds was 7.6 ± 0.7%, whereas only 1.5 ± 0.6% and 2.1 ± 0.8% of the ZUOErN3 and ZUOErN4 seeds, respectively, had germinated. After 57 h of imbibition, 36.4 ± 0.5% of the WT seeds had germinated, whereas the germination percentages of the ZUOErN3 and ZUOErN4 seeds were 25.5 ± 0.5% and 21.2 ± 0.8%, respectively. After 90 h of imbibition, no significant differences were observed in the overall germination rates of the WT and transgenic seeds, with all lines reaching over 95% germination (Fig. 1d). The above results showed that the overexpressing seeds germinated more slowly than the WT seeds, but no difference was observed in their overall germination rates.

### The delayed germination in *mOsNAC2*-overexpressing seeds is associated with an increased ABA content

Studies have shown that an increased ABA content in seeds slows germination, whereas a reduction in ABA content accelerates germination (Zhu et al., 2009). To investigate whether changes in the seed germination rates observed here were due to differences in the ABA levels, we applied different amounts of ABA to the WT seeds during germination. When an ABA concentration of 2.5 μmol/L was applied, the germination rate of the WT seeds decreased to a level similar to that of the transgenic seeds. On days 3, 5, and 7 after germination, the seedling growth potential was almost the same as the transgenic seedlings (Additional file 1: Fig. S1a–c). These results showed that the application of exogenous ABA to the WT seeds decreased their germination rate.

**Fig. 2:**
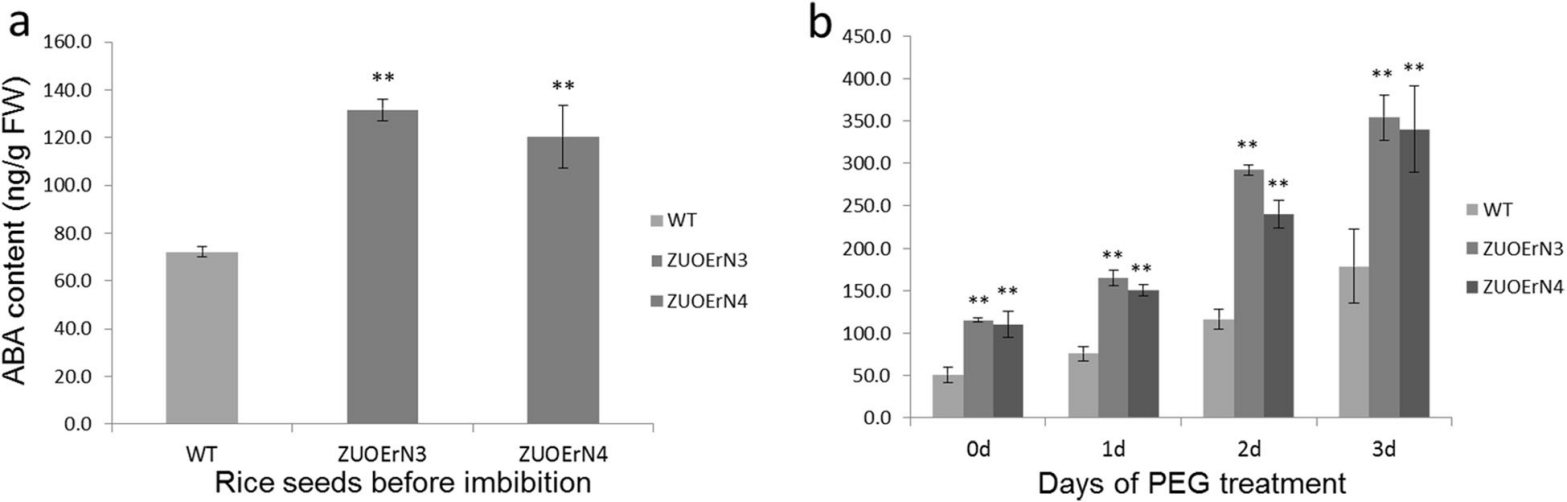
ABA levels are elevated in transgenic seeds and drought-treated seedlings. ABA levels in rice seeds (**a**) and seedlings with PEG treatment (**b**). Seedlings were cultured in hydroponic solution with 10% PEG 6000 to simulate drought. Error bars show the SD of three biological replicates. ** *P* < 0.01 (*t*-test)

ABA is a carotenoid-derived plant hormone. Fluridone (FLU) can inhibit the biosynthesis of carotenoids, and thus affects ABA biosynthesis (Yoshioka et al., 1998). When 80 μmol/L FLU was applied to the seeds during germination, the phenotypes of the WT and overexpressing plants were not significantly different after two days (Additional file 1: Fig. S2a, b); the ZUOErN3 and ZUOErN4 seedlings were 0.54 ± 0.15 cm and 0.55 ± 0.07 cm in length, respectively, while the WT seedlings were 0.53 ± 0.07 cm long. At three and five days after germination, the seedlings lengths were still not significantly different (Additional file 1: Fig. S2b; S3a, b); for example, the ZUOErN3 and ZUOErN4 seedlings were 2.11 ± 0.18 cm and 2.08 ± 0.21 cm in length, respectively, while the WT seedlings were 2.14 ± 0.31 cm long at three days after germination (Additional file 1: Fig. S2b). Thus, the inhibition of endogenous ABA biosynthesis results in a similar germination speed and seedling growth in the WT and overexpressing seeds. We then used the radioimmunoassay (RIA) method to investigate the endogenous ABA content in the seeds (Quarrie et al., 1988; Zhu et al., 2009). The ABA level in the WT seeds was 72.1 ± 2.2 ng/g, whereas it was significantly higher (131.6 ± 4.4 ng/g in ZUOErN3 and 120.4 ± 13.3 ng/g in ZUOErN4) in the transgenic seeds (Fig. 2a). This further supports the conclusion that increased ABA contents in the *mOsNAC2*-overexpressing plants delays germination.

### The *mOsNAC2*-overexpressing plants have higher drought tolerance

**Fig. 3:**
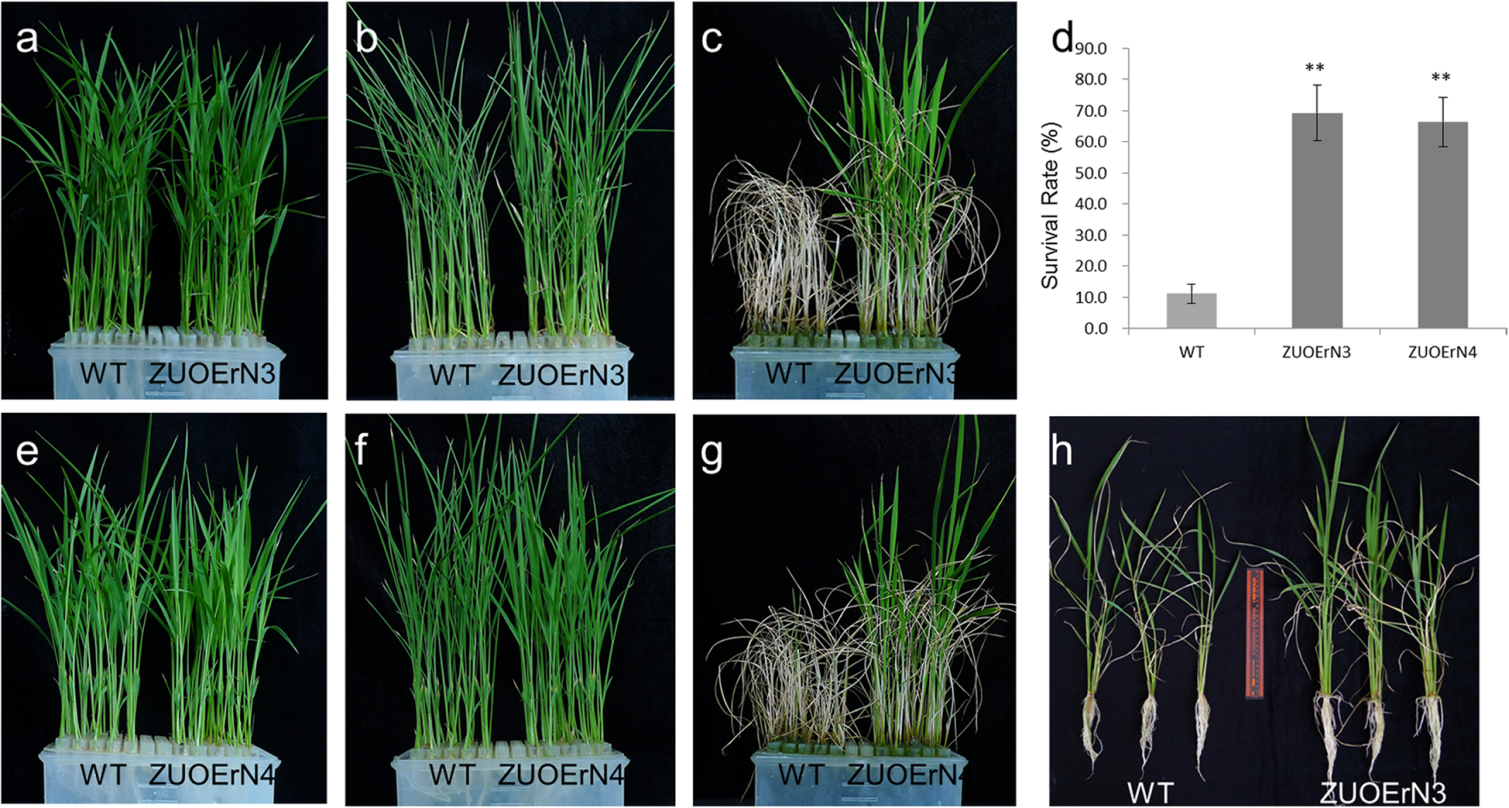
Drought tolerance of WT and *OsNAC2* overexpression plants. Seeds were germinated in water and transferred to Kimura B nutrient solution. Two-week-old seedlings were treated in Kimura B nutrient solution containing 10% PEG 6000. Plants before treatment (**a**, **e**), 10% PEG-treated for 3 d (**b**, **f**), and after 8 d treatment and then recovery for 8 d (**c**, **g**), are shown. **d**: The survival rate of WT and transgenic lines treated with 10% PEG 6000 in c and g. Error bars show the SD of three biological replicates (*n* = 60). ** *P* < 0.01 (*t-*test). **h**: The phenotype of WT and *OsNA*C2 overexpression line ZUOErN3 plants treated with 15% PEG 6000 for 5 d and allowed to recover for 7 d. Three independent experiments were performed. ZUONrE3, ZUOErN4: *OsNAC2* overexpression plants

Plant ABA contents are related to the tolerance of stresses such as drought (Zhu et al., 2009). We detected higher ABA contents in the *mOsNAC2*-overexpressing seeds (Fig. 2a); therefore, we explored whether the *mOsNAC2*-overexpressing plants have increased drought tolerance by subjecting two-week-old seedlings to polyethylene glycol (PEG)-induced drought conditions. The phenotypes of the WT, ZUOErN3, and ZUOErN4 seedlings were similar before PEG treatment (Fig. 3a, e), while after a three-day treatment, the leaves of the WT plants were curled and wilted, showing obvious symptoms of dehydration (Fig. 3b, f), whereas the leaves of ZUOErN3 and ZUOErN4 showed limited symptoms of drought stress (Fig. 3b, f). After an eight-day drought treatment and an eight-day recovery, most of the WT plants were shriveled and dead, while the overexpressing plants had green leaves (Fig. 3c, g). At the same time, the survival rates of the ZUOErN3 and ZUOErN4 plants were 69.2 ± 8.8% and 66.3 ± 7.9%, respectively, while that of the WT plants was only 11.2 ± 3.1% (Fig. 3d). These results showed that the overexpression of *OsNAC2* significantly increases the drought tolerance and survival rate at the seedling stage.

We further studied drought tolerance at the tillering stage. The plants were treated with 15% PEG for five days and allowed to recover for seven days, after which the transgenic plants appeared healthier than the WT. The transgenic plants grew new leaves, whereas the WT only recovered their green color (Fig. 3h). These results indicated that drought tolerance in the transgenic plants at the tillering stage is also higher than the WT.

### *mOsNAC2*-overexpressing plants have increased salt tolerance

**Fig. 4:**
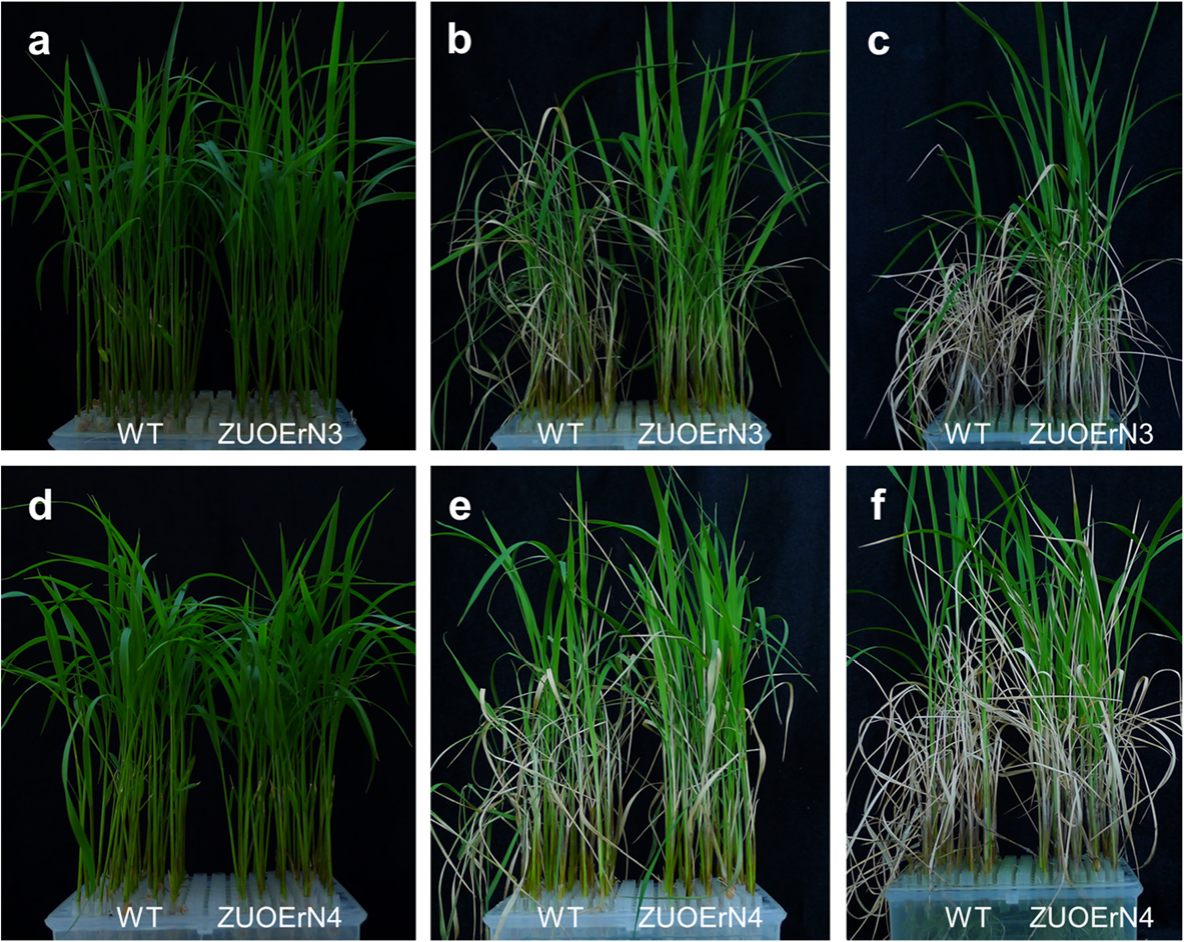
Salt tolerance of WT and *OsNAC2* overexpression plants. Seeds were germinated in water and transferred to Kimura B nutrient solution. Two-week-old seedlings were treated in Kimura B nutrient solution with 50 mM sodium chloride. WT and *OsNA*C2 overexpression plants (ZUONrE3 and ZUOErN4) before treatment (**a**, **d**), 50 mM sodium chloride treated for 8 d (**b, e**), and then recovery for 8 d (**c, f**) are shown

To study the response of the *mOsNAC2*-overexpressing plants to salinity stress, the seedlings were treated with 50 mM sodium chloride (salt). After eight days of treatment, most of the WT leaves were withered and dead, whereas only a few of the transgenic plants leaves had died, and new leaves had grown (Fig. 4a, b, d, e). After an eight-day recovery period, most of the WT plants were dead, whereas new leaves had grown in all the transgenic plants (Fig. 4c, f).

We explored the expression pattern of *OsNAC2* in the WT during salt stress responses using RT-qPCR. No obvious differences in the *OsNAC2* expression level were detected after a 1-h salt treatment, but the expression levels were greatly increased after 3 to 48 h of treatment (Additional file 1: Fig. S4). These results showed that overexpression of *OsNAC2* increased salinity tolerance in rice.

### ABA biosynthesis gene expression is increased in the *mOsNAC2*-overexpressing plants

**Fig. 5:**
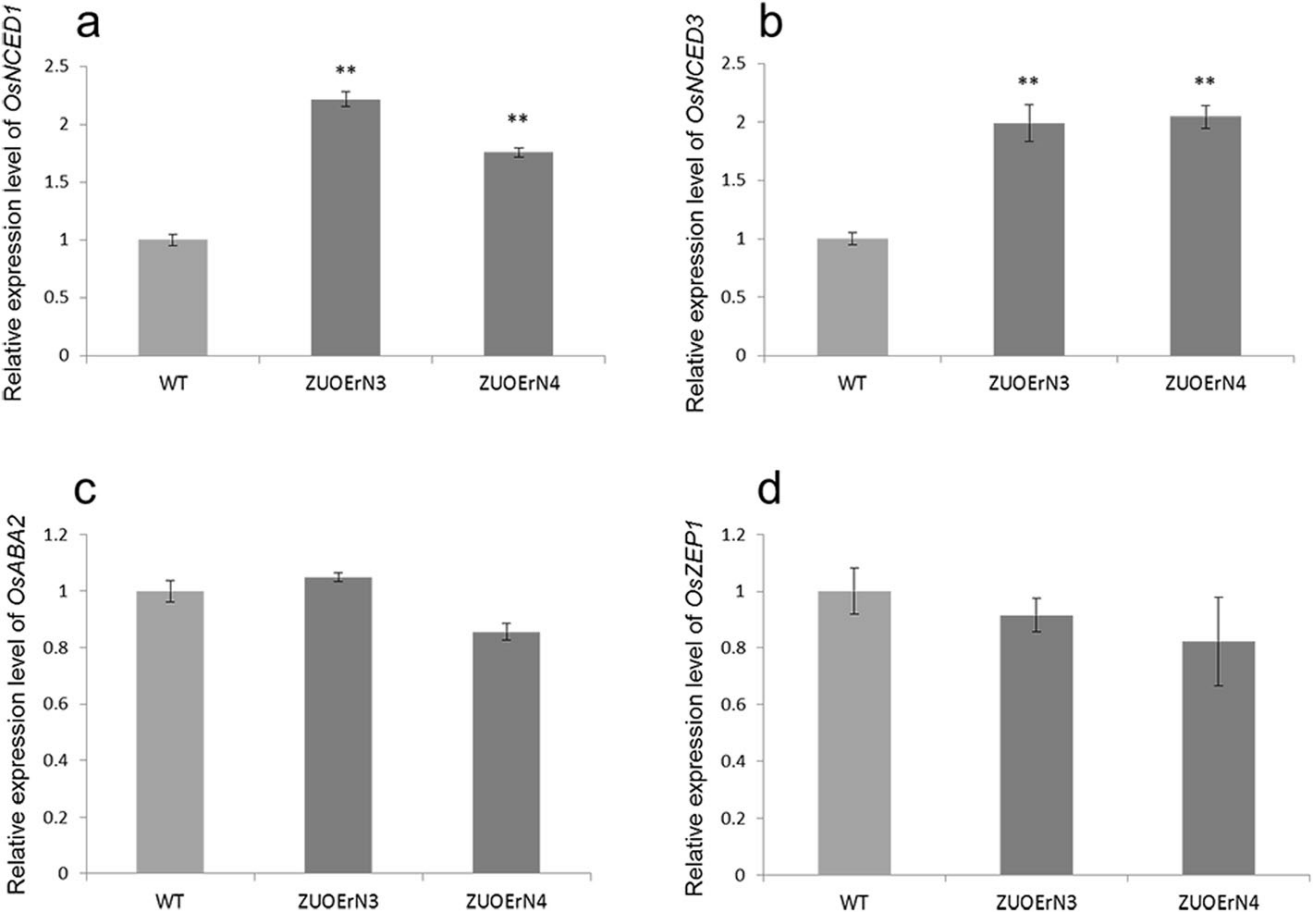
Expression levels of ABA biosynthesis-related genes. The relative expression levels of *OsNCED1* (**a**), *OsNCED3* (**b**), *OsABA2* (**c**), and *OsZEP1* (**d**) were assessed using RT-qPCR with leaves at the tillering stage. The data were normalized using the rice *UBI* gene and are shown relative to WT. Error bars represent SD of three biological replicates. ** *P* < 0.01 (*t*-test)

The nine-cis-epoxycarotenoid dehydrogenases (NCEDs) are the key enzymes in ABA biosynthesis (Zhu et al., 2009). *OsNCED1* is mainly expressed in the rice leaves under normal conditions (Ye et al., 2011). Our RT-qPCR analysis showed that *OsNCED1* was significantly more highly expressed in the transgenic plants than in the WT under normal growth conditions (Fig. 5a). *OsNCED3* is a major gene promoting ABA biosynthesis during drought stress in rice (Tan et al., 2003; Ye et al., 2011). Our RT-qPCR analysis revealed that the *OsNCED3* expression levels in the ZUOErN3 and ZUOErN4 lines were 0.99- and 1.04-fold higher than in the WT, respectively (Fig. 5b). We also analyzed the expression of genes encoding another two important enzymes in ABA biosynthesis, xanthoxin dehydrogenase (*OsABA2*) (Endo et al., 2014) and zeaxanthin epoxidase (*OsZEP1*) (Oliver et al., 2007); However, no significant differences were detected in the expression levels of either of these genes between the transgenic and WT plants (Fig. 5c, d). These results suggest that the increased expression of some ABA biosynthesis genes, such as *OsNCED1* and *OsNCED3*, may result in the higher ABA content observed in the transgenic plants.

### PEG increases the ABA content in the *mOsNAC2*-overexpressing seedlings

To determine whether ABA was involved in the enhanced drought tolerance observed in ZUOErN3 and ZUOErN4, the ABA concentrations were measured in PEG-treated seedlings. Before this drought treatment, the ABA contents in the transgenic (ZUOErN3 and ZUOErN4) and WT plants were 115.6 ± 2.5, 110.6 ± 15.1, and 51.0 ± 9.3 ng/g fresh weight (FW), respectively (Fig. 2b). After the PEG treatment, the ABA contents increased in both the transgenic and WT plants, although the ABA contents in the transgenic plants remained significantly higher than in the WT plants after 1-, 2-, and 3-day treatments (Fig. 2b). These results suggest that the higher ABA levels in transgenic plants may contribute to their increased drought tolerance.

### Stress-response genes are more highly expressed in the *mOsNAC2*-overexpressing plants

**Fig. 6:**
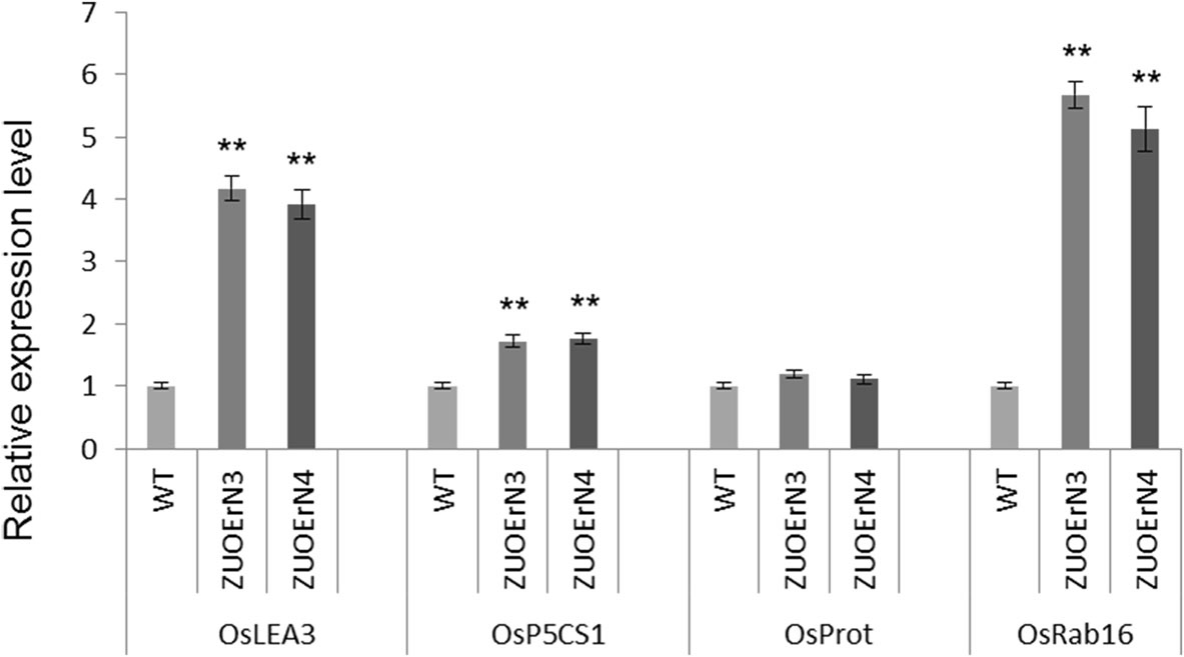
Expression level of abiotic stress-related genes. The relative expression levels of *OsLEA3*, encoding a group 3 late-embryogenesis abundant protein; *OsP5CS*, involved in biosynthesis of proline, encoding Δ1-pyrroline-5-carboxylate synthetase; *OsProt*, encoding a proline transporter; and *OsRab16*, encoding a small GTP-binding protein, were assayed using RT-qPCR with seedlings. The data were normalized using the rice *UBI* gene and are shown relative to WT. Error bars represent SD of three biological replicates. ** *P* < 0.01 (*t*-test)

To further investigate the possible mechanisms by which *OsNAC2* regulates the expression of stress-related genes, we employed RT-qPCR to evaluate the expression of several important abiotic stress-responsive genes in the WT and *mOsNAC2*-overexpressing lines. The expression of the drought-induced gene *OsLEA3* (encoding a group 3 late-embryogenesis abundant protein) was more than two-fold higher in the transgenic plants, while the expression of the ABA-responsive gene *OsRAB16* (encoding a small GTP-binding protein) was more than four-fold greater in these plants (Fig. 6). Proline is one of the most well-known osmoprotectants, and its accumulation has been widely observed in organisms under abiotic stress (Sripinyowanich et al., 2013). The expression of the proline biosynthesis gene *OsP5CS1* was about 0.7-fold higher in the transgenic plants compared with the WT, while the expression of *OsProt* did not significantly change (Fig. 6). These results suggest that *OsLEA3*, *OsRAB16*, and *OsP5CS1* are involved in the drought and salt stress responses.

### OsNAC2 directly regulates expression of *OsNCED3* and *OsLEA3*

**Fig. 7:**
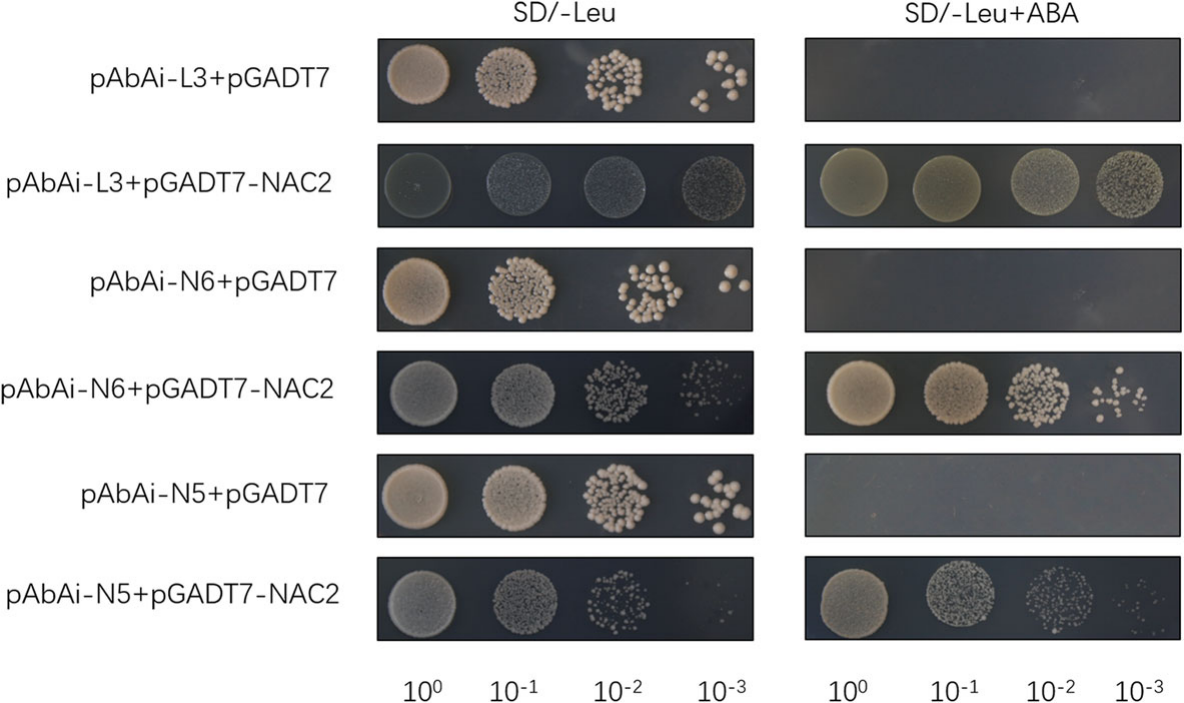
*OsLEA3* and *OsNCED3* are direct target genes of OsNAC2. The full-length *OsNAC2* cDNA was fused to the *GLA4* activation domain in the prey vector. The promoter sequence regions of different lengths were fused to an AbAi reporter gene in the bait vector. For yeast one-hybrid assay, Yeast cells were cotransformed with a bait vector and a prey vector and grown in liquid medium and diluted in a 10× dilution series (from 10^0^ to 10^− 3^). Each dilution was spotted on both SD/−leu and SD/−leu with 300 ng/ml AbA to suppress the background and to test the strength of the interaction. L3, partial *OsLEA3* promoter sequence; N6 and N5, partial *OsNCED3* promoter sequences

In this study, the expression levels of *OsNCED3* and *OsLEA3* were significantly increased in the *mOsNAC2*-overexpressing seedlings than in WT. We speculate that these two genes may be targets of OsNAC2. To further investigate the candidate targets of OsNAC2, we performed yeast one-hybrid assays to test the interaction of OsNAC2 with the promoters of *OsLEA3* and *OsNCED3*. The full-length *OsNAC2* cDNA was fused in-frame to the GAL4 activation domain in the pGADT7 vector. Promoter sequence regions of different lengths were ligated into the pAbAi vector. The yeast one-hybrid assays showed that OsNAC2 directly interacts with the promoters of *OsLEA3* and *OsNCED3* (Fig. 7).

## Discussion

NAC TFs play important functions in rice stress tolerance. In this study, we identified *OsNAC2* as a positive regulatory factor of abiotic-stress tolerance, as the *mOsNAC2*-overexpressing rice plants exhibited an increased tolerance to drought and salt stress. Previous studies have shown that the expression levels of *SNAC1* (*STRESS-RESPONSIVE NAC1*) and *OsNAC6* are induced by drought and salinity, and that transgenic rice plants overexpressing these genes also display increased drought and salt tolerance (Hu et al., 2006; Lee et al., 2017). Taken together, the results of these studies and our present work point to similar roles for the NAC TFs in abiotic stress responses. In addition, our previous study revealed that *mOsNAC2*-overexpressing plants have an ideotype plant architecture, with thick stems, well-developed root systems, and high yields (Jiang et al., 2018). A highly developed root system can absorb more moisture and nutrients from the soil, thereby conferring better drought tolerance and facilitating higher yields (Henry et al., 2012). Several other NAC TFs can increase rice yields under abiotic stress conditions (Jeong et al., 2010, 2013). *OsNAC10* increases rice yields under stress conditions. *OsNAC5*-overexpressing rice also enhanced drought tolerance and increased grain yields under drought conditions. OsNAC5 interacts with *OsLEA3*, an abiotic stress-induced gene in rice that plays vital role in drought and salt tolerance (Jeong et al., 2013), similar to the direct interaction of OsNAC2 with the promoter of *OsLEA3* observed in our current study. We also found the miR164b-resistant *mOsNAC2*-overexpressing plants to have enhanced tolerance of drought and salt stress, partly because of their direct regulation of *OsLEA3*.

ABA plays an important role in the regulation of stress responses in plants. Many genes in the *NAC* gene family can increase stress tolerance, which is intimately associated with ABA (Chen et al., 2014; Huang et al., 2016; Hong et al., 2016; Shen et al., 2017). In this study, the *mOsNAC2*-overexpressing plants exhibited an increased tolerance of drought and salt, and further measurements showed that their ABA contents were significantly higher than in the WT. The expression levels of *OsNCED1* and *OsNCED3*, encoding rate-limiting enzymes in ABA biosynthesis, were also significantly higher in the transgenic plants than in the WT. These findings suggest that *OsNAC2* regulates the expression of ABA biosynthesis genes to increase the ABA content, thereby increasing abiotic-stress tolerance. It was previously reported that OsNAC2 directly promotes the expression of *OsNCED3* to increase the ABA content (Mao et al., 2017). We also illustrated that OsNAC2 directly interacts with the promoter of *OsNCED3* in the yeast one-hybrid system, supporting the results of the previous report.

The expression levels of several abiotic stress-responsive genes were significantly increased in the *mOsNAC2*-overexpressing plants compared with the WT. These genes included *OsLEA3*, encoding a group 3 late-embryogenesis abundant protein (Xiao et al., 2007); *OsRAB16*, encoding a small GTP-binding protein (Yong et al., 2009); and *OsP5CS1*, encoding Δ1-pyrroline-5-carboxylate synthetase, an enzyme involved in the biosynthesis of proline (Sripinyowanich et al., 2013). This increased expression suggests that the enhanced stress tolerance in the *mOsNAC2*-overexpressing plants may also be related to the proline levels and these candidate proteins, which mediate abiotic stress tolerance. Indeed, it has been reported that proline accumulation is a possible indicator of osmotic stress tolerance, as it participates in regulation of the osmotic pressure of the cytoplasm in plants (Do et al., 2003). In addition, Rab family proteins play crucial roles in the plant tolerance of environmental stresses. *OsRAB16* is an ABA-responsive gene (Mohamed and Aisha, 2019) and exhibited greater than four-fold higher expression in the *mOsNAC2*-overexpressing plants than in the WT, consistent with the increased ABA levels observed in the transgenic plants. Finally, OsNAC5 directly interacts with the promoter of *OsLEA3* to regulate salt tolerance (Jeong et al., 2013); here, we showed that OsNAC2 directly interacts with the promoter of *OsLEA3* to regulate drought and salt tolerance.

NAC TF proteins have important functions in the growth and development of rice, with each NAC TF playing many roles; for example, the NAC TF OsNAP regulates leaf senescence (Liang et al., 2014) and increases abiotic stress tolerance during the vegetative and reproductive stages (Chen et al., 2014). *OsNAC5* and *OsNAC10*, when specifically expressed in the roots, can modify root structure and increase drought tolerance, thereby increasing yields under drought conditions (Jeong et al., 2010, 2013). Our study, along with others, indicates that *OsNAC2* has several important functions in the growth and development of rice, especially in enhancing yields (Jiang et al., 2018). Here, we have shown that *OsNAC2* plays a positive regulatory role in drought- and salt-tolerance in rice through ABA-mediated pathways. These findings may enable the production of new stress-tolerant and high-yielding rice varieties.

## Conclusions

Our results demonstrated that OsNAC2 positively regulates rice tolerance of drought and salt stress via an ABA-mediated pathway. This study increases our understanding of the molecular mechanisms by which rice responds to drought and salt stress and broadens our knowledge of NAC TF function in rice. This information will be helpful for breeding drought- and salt-tolerant rice varieties using genetic engineering.

## Methods

### Rice germination and growth conditions

Seeds of rice (*Oryza sativa* L.) WT and *mOsNAC2*-overexpressing lines were harvested during the same season. The seeds were imbibed for 2 d in water with or without 80 μmol/L FLU or 2.5 μmol/L ABA at 25 °C/23 °C (day/night) in a growth chamber. After four days, the germinated seeds were transferred into Kimura B complete nutrient solution (Yoshida et al., 1976) under photosynthetically active radiation 600–1500 μmol m^− 2^ s^− 1^. The two-week-old seedlings were used for further experiments.

### PEG and salt treatments

For salt stress conditions, 2-week-old seedlings were treated with Kimura B complete nutrient solution supplemented with 100 mM sodium chloride and maintained in a greenhouse with a 13/11-h day/night cycle (25/23 °C). After stress treatments, the seedlings were harvested separately at 0-, 1-, 3-, 6-, 12-, 24-, and 48-h intervals. The tissues were frozen in liquid nitrogen and stored at − 80 °C until RNA extraction.

To analyze drought tolerance of the transgenic plants, WT and transgenic homozygous seeds (ZUOErN3 and ZUOErN4) were germinated in water and transferred to Kimura B nutrient solution 4 days after germination. The 2-week-old seedlings were then transferred into new Kimura B nutrient solution with 10% PEG 6000. The plants were photographed before treatment, at 3 d, and at 8 d, and then following an 8-d recovery period, during which they were treated with Kimura B nutrient solution only. The survival rate was measured after seedling recovery. PEG-treated seedlings, simulating drought treatment, were collected after treatment in the 10% PEG solution for 0 h, 1 d, 2 d, and 3 d, and stored at − 80 °C for ABA determination.

To analyze salt stress, the seedlings were treated with Kimura B nutrient solution with 50 mM sodium chloride. The plants were photographed before treatment, after treatment for 8 d, and after recovery for 8 d.

### Germination treatment

Rice seeds were imbibed in water for 2 d. The seeds were then directly sown in petri dishes on filter paper to allow determination of percentage germination. To observe the effects of ABA and fluridone (FLU) on germination, 2.5 μmol/L ABA and 80 μmol/L FLU were added to the water before imbibition. Seeds were placed in a growth chamber with a 13/11-h day/night cycle (25/23 °C) to facilitate germination. The germination rate was recorded or photographed, depending on the experiments. Each experiment was repeated three times.

### Measurement of the endogenous ABA level

For estimation of the endogenous ABA level in seeds and seedlings, 0.5 g of rice seeds or 1.0 g of seedlings was homogenized in 3 ml of distilled water and then shaken at 4 °C overnight. The homogenates were centrifuged at 12,000 g for 10 min at 4 °C and the supernatants used for an RIA assay. The subsequent steps were conducted as previously described (Quarrie et al., 1988; Zhu et al., 2009).

### RNA isolation and RT-qPCR

RNA was isolated using an RNA extraction kit with TRIzol reagent (Invitrogen, USA) and quantified with a DU730 spectrophotometer (Beckman Coulter, Germany). cDNA was synthesized with a 5 × iScript RT Supermix kit (TaKaRa, Dalian, China) using about 2 μg of total RNA. RT-qPCR was performed with SYBR premix Ex Taq II in a total volume of 20 μL on the Bio-Rad CFX 96 following the manufacturer’s protocol. Data were normalized to the internal rice *UBIQUITIN* (*UBI*) gene, and relative quantification was used for data analysis.

The primers for rice *UBI* and *OsNAC2* amplification are described in Jiang et al. (2018). The primers for *OsNCED1* and *OsNCED3* are described in Zhu et al. (2009). The primers for *OsLEA3*, *OsP5C91*, *OsProt*, and *OsRab16* are described in Yin et al. (2017). The primers used for *OsABA2* and *OsZEP1* are as follows: ABA2F, 5′-TGTGGATCTGCTACCTAAGG-3′; ABA2R, 5′-GTAAAGCCACCATCCACCATG-3′; ZEPF, 5′-CGGATGCCATTGAGTTTGGTTC-3′; and ZEPR, 5′-CAGATTCATATGGGAGCGTGC-3′.

### Yeast one-hybrid

For yeast one-hybrid assays, the The full-length *OsNAC2* cDNA was fused in-frame to the *GAL4* activation domain in the pGADT7 vector. The different promoter sequences regions of *OsNCED3* and *OsLEA3* were ligated into the pAbAi vector. The assays were performed according to the manufacturer’s instructions (CLONTECH Laboratories, USA; cat. # 630491) or the reported publication (Xie et al., 2018). The L3, partial *OsLEA3* promoter sequence; N6 and N5, partial *OsNCED3* promoter sequences were followed the previous publication (Jeong et al., 2013; Mao et al., 2017).

## Supplementary information

**Additional file 1: Figure S1.** Effects of ABA on rice seed germination. WT seeds were imbibed in water containing 2.5 μmol/L ABA at 25/23 °C (day/night). Seeds from *mOsNAC2*-overexpressing lines (ZUOErN3 and ZUOErN4) were imbibed in water only. Images were taken after germination for 3 d (a), 5 d (b), and 8 d (c). **Figure S2.** Effects of fluridone (FLU) on rice seed germination. WT seeds were imbibed in water at 25/23 °C (day/night). Seeds from *OsNAC2* overexpression lines (ZUOErN3 and ZUOErN4) were imbibed in water containing 80 μmol/L FLU at 25/23 °C (day/night). The images were taken on day 2 after germination (a). The seedling length was measured after germination for 2 d, 3d, and 5 d (b). **Figure S3.** Effects of FLU on rice seedling growth. WT seedlings were cultured in water at 25/23 °C (day/night). *OsNAC2* overexpression lines (ZUOErN3 and ZUOErN4) were cultured in water containing 80 μmol/L FLU. The images were taken on day 3 (a) and day 5 (b) after germination. **Figure S4.** Expression level of *OsNAC2* under salt stress. The expression of *OsNAC2* in rice seedlings grown in 50 mM sodium chloride was assayed by RT-qPCR. RT-qPCR data were normalized using the rice *UBI* gene and are shown relative to 0 h. Error bars represent SD of three biological replicates.

## Data Availability

All data supporting the conclusions of this article are provided within the article (and its additional files).

## Abbreviations

ABA: Abscisic acid
ABA2: xanthoxin dehydrogenase
FLU: fluridone
LEA3: 3 late-embryogenesis abundant protein
miRNA: microRNA
NAC: NAM, AFAT, and CUC transcription factor
NCEDs: Nine-cis-epoxycarotenoid dehydrogenases
P5CS1: Δ1-pyrroline-5-carboxylate synthetase
PEG: Polyethylene glycol
RAB16: a small GTP-binding protein
RIA: Radioimmunoassay
RT-qPCR: Reverse transcription quantitative PCR
WT: wild-type
ZEP1: Zeaxanthin epoxidase
