# Supplementary figures and images for: Overexpression of a microRNA-targeted NAC transcription factor improves drought and salt tolerance in Rice via ABA-mediated pathways
Source: Rice (N Y). 2019 Oct 21;12:76. doi: 10.1186/s12284-019-0334-6 (PMC6803609; doi:10.1186/s12284-019-0334-6)

S Fig. 1

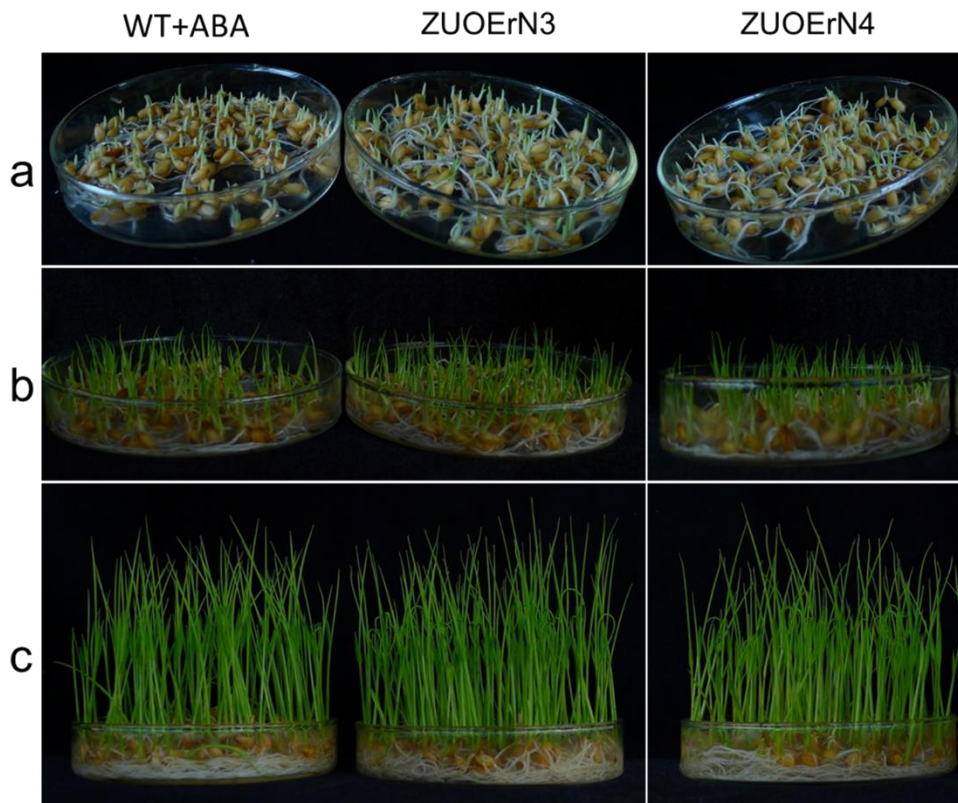

S Fig. 2

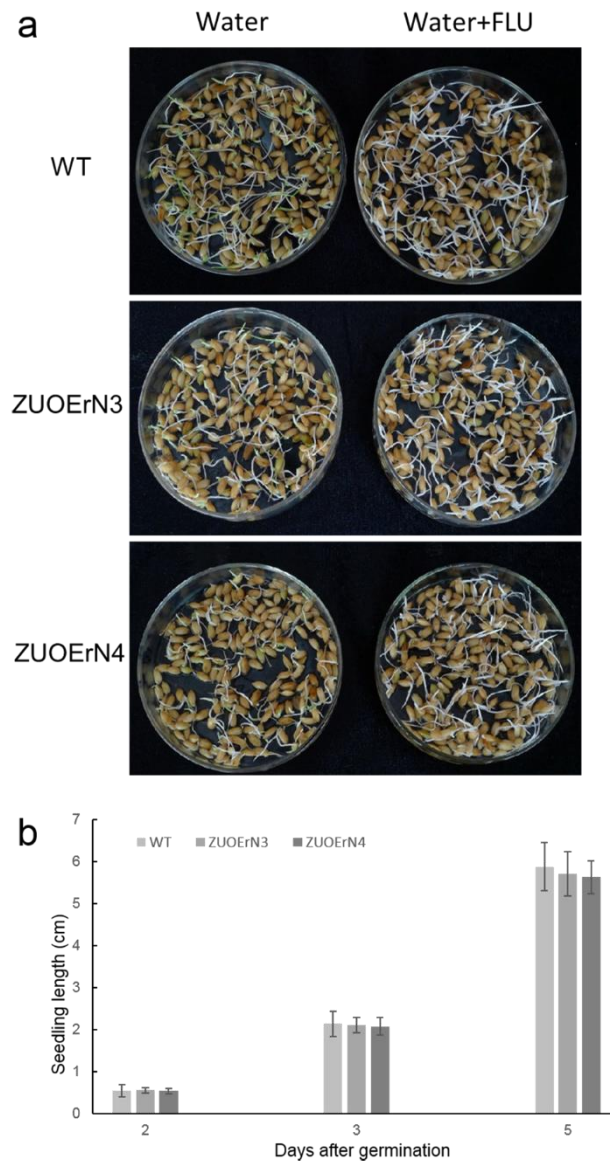

S Fig. 3

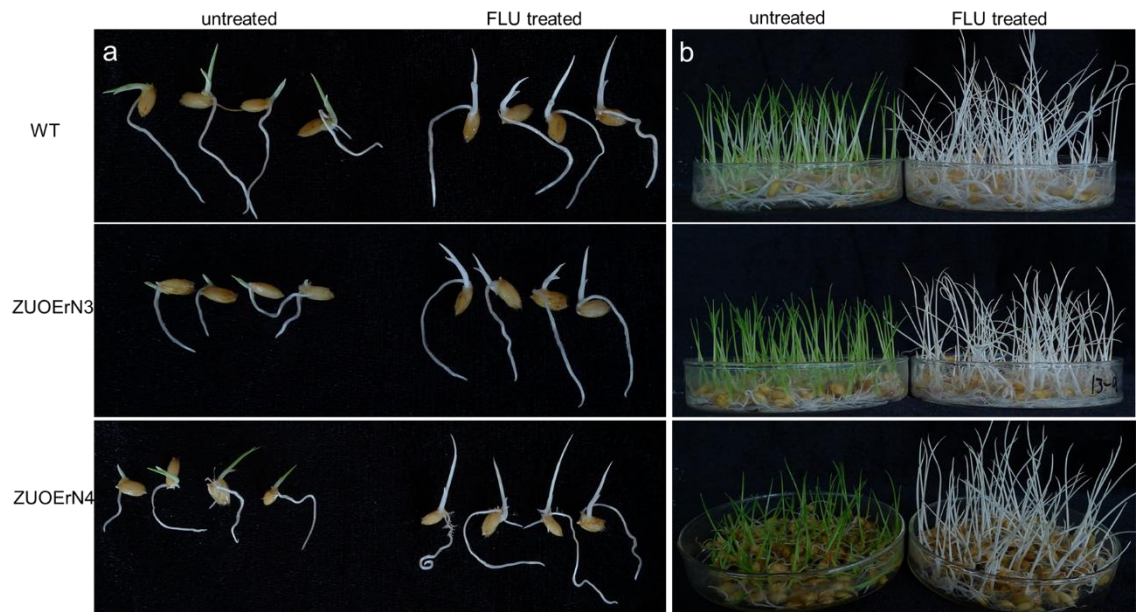

S Fig. 4

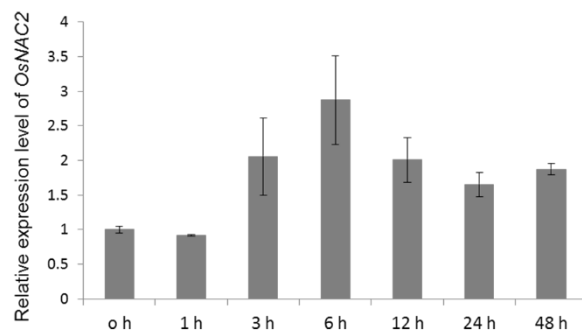

Supplement: Supplementary file 1 — Additional file 1: Figure S1. Effects of ABA on rice seed germination. WT seeds were imbibed in water containing 2.5 μmol/L ABA at 25/23 °C (day/night). Seeds from mOsNAC2-overexpressing lines (ZUOErN3 and ZUOErN4) were imbibed in water only. Images were taken after germination for 3 d (a), 5 d (b), and 8 d (c). Figure S2. Effects of fluridone (FLU) on rice seed germination. WT seeds were imbibed in water at 25/23 °C (day/night). Seeds from OsNAC2 overexpression lines (ZUOErN3 and ZUOErN4) were imbibed in water containing 80 μmol/L FLU at 25/23 °C (day/night). The images were taken on day 2 after germination (a). The seedling length was measured after germination for 2 d, 3d, and 5 d (b). Figure S3. Effects of FLU on rice seedling growth. WT seedlings were cultured in water at 25/23 °C (day/night). OsNAC2 overexpression lines (ZUOErN3 and ZUOErN4) were cultured in water containing 80 μmol/L FLU. The images were taken on day 3 (a) and day 5 (b) after germination. Figure S4. Expression level of OsNAC2 under salt stress. The expression of OsNAC2 in rice seedlings grown in 50 mM sodium chloride was assayed by RT-qPCR. RT-qPCR data were normalized using the rice UBI gene and are shown relative to 0 h. Error bars represent SD of three biological replicates. [file 12284_2019_334_MOESM1_ESM.pdf]
